# Broadband synchronization of ultrafast pulse generation with double-walled carbon nanotubes

**DOI:** 10.1515/nanoph-2023-0192

**Published:** 2023-07-24

**Authors:** Jiancheng Zheng, Diao Li, Peng Liu, Xiaoqi Cui, Bin Zhang, Wei Geng, Qiang Zhang, Zhenyu Xu, Esko I. Kauppinen, Zhipei Sun

**Affiliations:** QTF Center of Excellence, Department of Electronics and Nanoengineering, Aalto University, FI-00076 Espoo, Finland; Department of Applied Physics, Aalto University, FI-00076 Espoo, Finland; College of Information Engineering, SanmingUniversity, Sanming 365004, China

**Keywords:** double-walled carbon nanotube, fiber laser, mode-locking, synchronization

## Abstract

Double-walled carbon nanotubes have shown competitive properties in broadband optical pulse generation owning to the intrinsic electronic properties. Synchronization of ultrafast optical pulses in multiple wavelengths is a key technique for numerous applications, such as nonlinear frequency conversion, ultrafast pump-probe, coherent Raman scattering spectroscopy, coherent optical synthesis, etc. In this work, we demonstrate the mode-locking and synchronization of 1.55 µm pulses with 1 µm and 1.9 µm pulses via a single saturable absorber based on double-walled carbon nanotubes. The large optical nonlinearity and broadband optical absorption in the double-walled carbon nanotubes enable independent and synchronized mode-locking in >900 nm bandwidth. In addition, we present a creative concept to realize multi-wavelength synchronization from a single laser system. Our results demonstrate a straightforward and feasible approach towards pulse synchronization over ultra-broad bandwidth with flexible wavelength selection in the near-infrared region.

## 1 Introduction

The rapid development of ultrafast pulse generation technologies has spurred great advancements in science and applications. As an extension of single wavelength sources, dual-wavelength synchronized ultrashort optical pulses with a large wavelength difference and superior stability are important tools for numerous applications in nonlinear frequency conversion, ultrafast pump-probe, coherent Raman scattering spectroscopy, coherent optical synthesis, and many other timing and wavelength dominated nonlinear optics [1–4]. The synchronization of dual-wavelength ultrafast pulses has been intensively investigated since the invention of Ti:sapphire lasers thanks to their ultrabroad homogeneous gain spectrum and easy self-mode-locking by Kerr lens medium. Ingenious configuration design allows the oscillation of dual-wavelength pulses in a single or double-cavity with a shared Ti:sapphire rod, where mode-locked synchronization was achieved by tuning the dispersion or moving the reflection mirror position [5–7]. Despite a second gain module is not required in these methods, the two laser beams need to overlap in the gain medium for self-synchronization, which induces instability when gain competition arises in the case the overlap mode volume is large [8]. Modified approaches were proposed later via introducing active feedback to the oscillators or simply by using two separate gain media (e.g., Ti:sapphire and Nd:YVO_4_ or Ti:sapphire and Cr:forsterite) in the synchronized lasers [9, 10], while both add more complexity to the solid-state laser system.

In contrast, fiber lasers that adopt rare earth elements as optical gain and amplification media enable the coverage of a large wavelength range at the infrared region and provide other inherent advantages, such as compactness, free optical alignment, and large optical nonlinearity [11, 12]. In principle, it is not difficult to synchronize dual-wavelength pulses in fiber if stable mode-locking is obtained in two resonators. The key criterion is to simultaneously lock the modes over a broadband wavelength range with an appropriate modulator. During the past decades, semiconductor saturable absorber mirrors (SESAMs) have been well developed for passive mode-locking [13], which is advantageous to the availability of controllable parameters for different types of lasers (e.g., solid-state lasers, semiconductor lasers, fiber lasers) [14–16], but is usually limited in operation bandwidth for a single device. For instance, dual-wavelength pulse synchronization in ytterbium and erbium fiber lasers has been demonstrated either by optical injection method (i.e., master–slave synchronization) or in a shared fiber cavity configuration (i.e., coupled synchronization) [17, 18], where different SESAM devices designed for 1 µm and 1.55 µm wavelengths are employed to mode-lock them separately.

Recently, the rapid progress in nanomaterials research has promoted new solutions to facilitate the mode-locking in fiber lasers. Representative works are the demonstrations of using single-walled carbon nanotubes and graphene as self-modulation devices (i.e., saturable absorbers) [19–22]. Compared with single-walled carbon nanotubes, where the characteristic absorption peaks corresponding to their operation wavelengths need to be tailored by mainly careful control of the nanotube diameter distribution during the material synthesis, the relatively flat spectrum absorption of double-walled carbon nanotubes (DWCNTs) contributed by the inner and outer tubes’ excitonic transition energies can be a better alternative in multiple-wavelength laser operations [23, 24].

In this work, we present a directly coupled fiber laser configuration mode-locked with a shared DWCNT saturable absorber for three wavelengths, which are emitted by the ytterbium, erbium, and thulium ions doped in different gain fibers. In one setup, an erbium cavity is employed to couple with a ytterbium cavity for the synchronization of pulses at 1 and 1.55 µm wavelengths. In another setup, an erbium cavity is used to couple with a thulium cavity to synchronise pulses at 1.55 µm and 1.9 µm wavelengths. The same DWCNT saturable absorber is utilized in these two setups to mode-lock the three wavelengths with a maximum spectral space of up to ∼900 nm. In both synchronisation cases, the DWCNT saturable absorber film is positioned at the common section of the laser resonators, in which pulse attraction induced by the cross-phase modulation provides a self-motivated mechanism for the synchronization of two pulses at different wavelengths. The use of a variable optical delay line in the erbium cavity permits high precision round trip frequency (repetition rate) tuning of the 1.55 µm laser. Experimental results find that the self-synchronization forms and the dual-wavelength pulses oscillate in the same repetition rate when one laser optical cavity length is tuned approach to the other. Our DWCNT sample performs outstanding resistance to potential thermal and optical damages, as demonstrated by the superior stability of the synchronized operation during a few hours of continual surveillance.

## 2 Material synthesis and characterization

**Figure 1:**
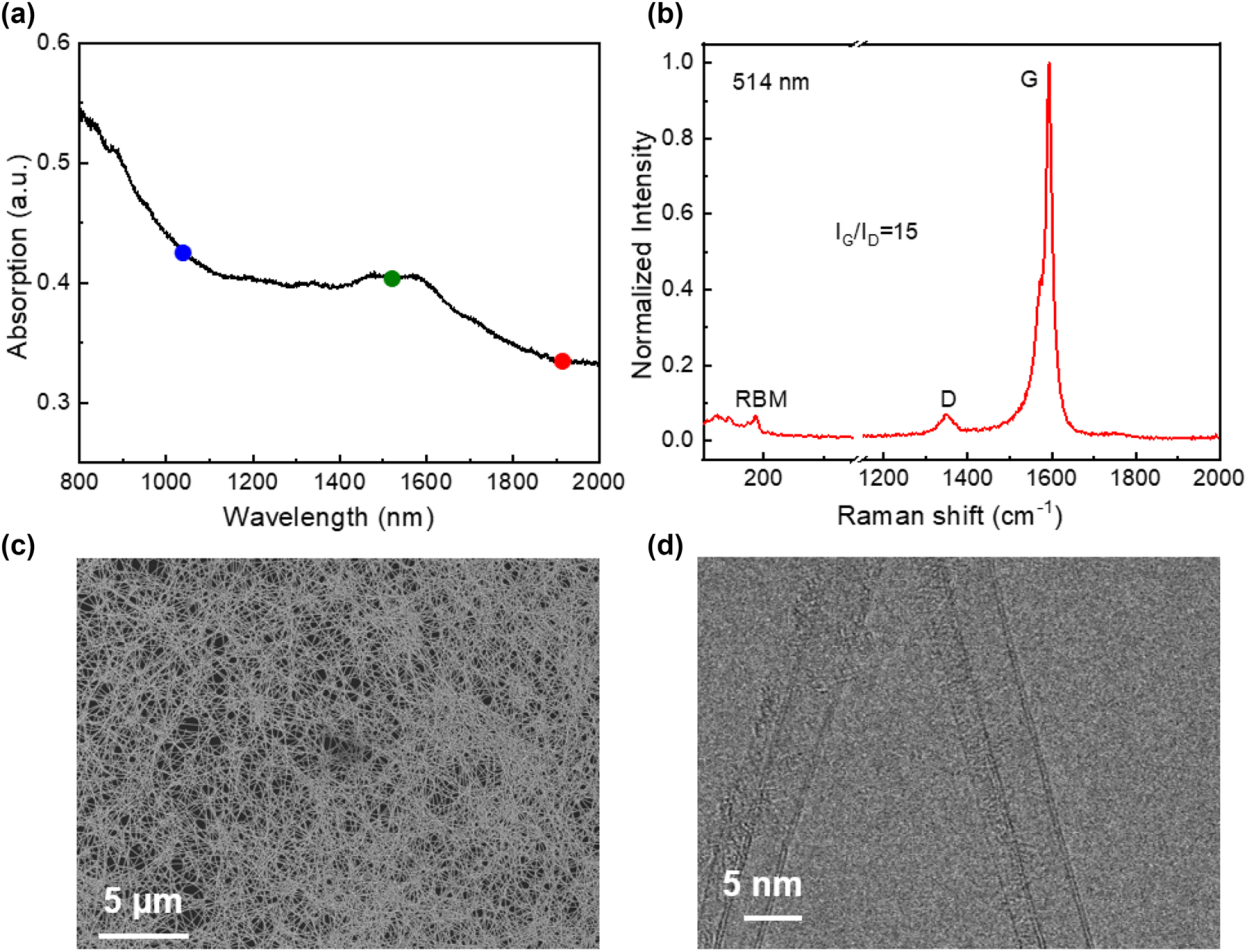
Material characterization of the DWCNT film. (a) Broadband optical absorption spectrum of the DWCNT film (The blue, green, and red disks indicate the 1 µm, 1.55 µm, and 1.9 µm wavelengths); (b) Raman spectroscopy of the synthesized DWCNT film excited by a 514 nm laser; (c) SEM; and (d) TEM images of the synthesized DWCNT film.

The pristine DWCNTs were synthesized by floating catalyst chemical vapor deposition method in a vertically placed tube furnace. The furnace was heated to 1100 °C to satisfy the chemical reaction temperature, in which CH_4_ and gas-phase precursors, including ferrocene, sulfur and carrier gases (N_2_ and H2), were utilized to synthesize DWCNTs in the reactor [25]. The covalently bonded DWCNTs were synthesized by a gas-phase reaction in the hot zone of reactor. The aerosol-like nanotubes were easily collected by a large-area membrane filter at room temperature, and the film thickness could be precisely controlled by the collection time. Figure 1(a) shows the broadband optical absorption spectrum of the DWCNT film measured by an ultraviolet–visible–near infrared spectrometer (Agilent Cary 5000). The large absorption span enables it a potential application for ultrafast pulse generation with working wavelengths ranging from ∼1 μm to 2 μm. The Raman spectrum of the DWCNTs excited by a 514 nm laser (Horiba Jobin-Yvon Labram HR 800) is shown in Figure 1(b). Obvious peaks in the Raman shift at 190 cm^−1^ are observed from the radial breathing mode (RBM), which corresponds to the formation of the inner tube in the DWCNT film. The dominant peak located at ∼1594 cm^−1^ is a graphite-like (G) band, and the peak located at ∼1351 cm^−1^ is the D band, referring to the disordered properties originating from the vacancies or any defects. The intensity ratio of G/D is widely used to evaluate the quality of nanotubes. Here the ratio is as high as 15, indicating the good quality of DWCNTs. To investigate the nanostructure and morphology of the DWCNTs, scanning electron microscopy (SEM, Zeiss Sigma VP) and Cs-corrected transmission electron microscopy (TEM, JEM2200FS, JEOL Ltd) are used to observe the microstructures of the DWCNTs. The SEM image in Figure 1(c) shows a uniform network structure formed by the disordered stacking of DWCNTs. In addition, CNTs with a diameter of about ∼5 nm were observed in Figure 1(d), which shows the obvious double-walled structure in our sample.

## 3 Experimental setup

**Figure 2:**
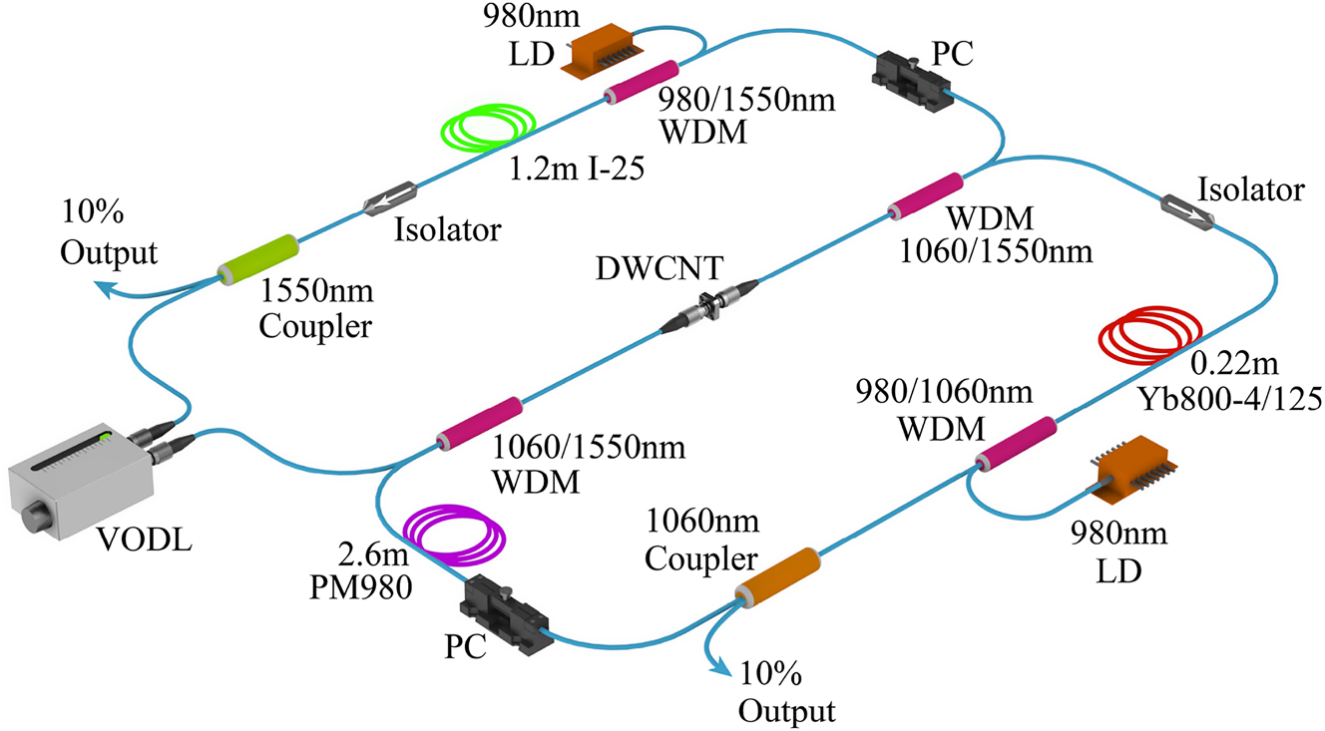
Experimental setup of the dual-wavelength fiber laser. LD, laser diode; WDM, wavelength division multiplexer; PC, polarization controller; VODL, variable optical delay line; DWCNT, double-walled carbon nanotube. The fiber rings in green, red, and purple represent the erbium-doped fiber, ytterbium-doped fiber, and polarization-maintaining fiber.

The experimental setup of the dual-wavelength fiber laser is schematically shown in Figure 2. We construct a composite fiber cavity with ytterbium-doped fiber and erbium-doped fiber for the synchronisation of pulses at ∼1 µm and 1.55 µm wavelengths, which are managed in two fiber loops bridged by a common branch. The broadband DWCNT film coated on the fiber end is placed in the common fiber to work as a joint saturable absorber in the composite cavity. The two individual wavelength lasers are combined and divided by two 1060/1550 nm wavelength division multiplexers (WDMs). Couplers (with 90:10 ratio, 10 % output), polarization-independent isolators and polarization controllers (PCs) working at 1 µm and 1.55 µm wavelengths are incorporated into each resonator to extract the intracavity light, start the mode-locking and ensure unidirectional operation, respectively. A segment of 1.2 m erbium-doped fiber (Fibercore I-25) is used for the telecom band emission at ∼1.55 µm, which is pumped by a 976 nm laser diode via a 980/1550 nm WDM. The ytterbium-doped fiber is pumped by another 976 nm laser diode via a 980/1060 nm WDM. High-gain ytterbium-doped fiber (Yb800-4/125, 0.22 m) with 800 dB/m absorption at 975 nm is employed for the mission at ∼1 µm. A section of ∼2.6 m polarization-maintaining fiber (Thorlab PM980) is added to the 1 µm laser to form a fiber-based Lyot filter for intra-cavity spectral filtering, which facilitates the start of mode-locking in the all-normal-dispersion resonator [26]. A variable optical delay line (OZ OPTICS ODL-650) is employed in the 1.55 µm laser resonator to adjust the optical length. The total length of each oscillator is approximately 10 m. The output performance of the dual-wavelength laser is assessed by a power meter (Thorlabs PM100D), an optical spectrum analyzer (Anritsu MS9740A), an autocorrelator (APE PulseCheck 150), a digital oscilloscope (Siglent SDS5104X), and a radio frequency spectrum analyzer (Anritsu MS2692A) assisted by an ultrafast photodiode detector (EOT ET-5000F).

## 4 Synchronization of 1.55 µm laser with 1 µm laser

**Figure 3:**
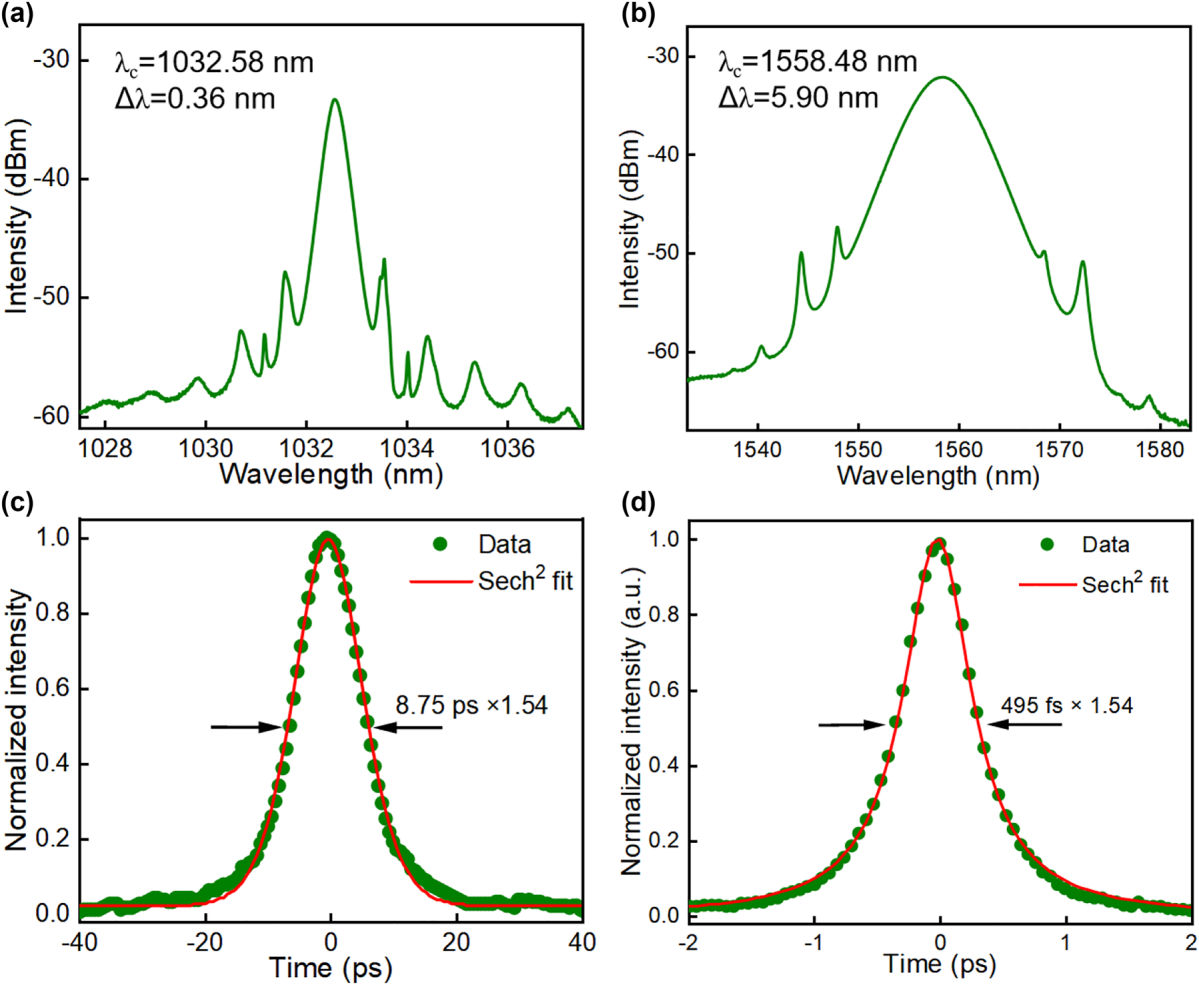
Synchronized lasers at 1 and 1.55 µm. (a) 1 µm ytterbium laser output soliton spectrum; (b) 1.55 µm erbium laser output soliton spectrum; (c) 1 µm ytterbium laser pulse autocorrelation trace; (d) 1.55 µm erbium laser pulse autocorrelation trace.

Independent fundamental mode-locking is observed in the ytterbium laser and erbium laser when their pump powers are in the range of 61.8–67.9 mW and 22.5–28.5 mW, respectively. Further increasing the pump power will turn the fundamental frequency pulse to multiple pulses rather than material damage, which benefits our nanotube robustness against optical intensity. The initial mismatch of the ytterbium cavity and the erbium cavity is a few centimeters, which can be compensated by tuning a variable optical delay line placed in the erbium cavity. Synchronized mode-locking is obtained when the two cavities’ lengths are approximately equal via reducing the optical delay in the erbium cavity. Mode-locked laser spectra were measured from the 10 % output couplers in both cavities, as shown in Figure 3(a) and (b). The synchronized pulses are mode-locked at the central wavelength of ∼1032.58 nm, with ∼0.36 nm bandwidth in the ytterbium laser, and ∼1558.48 nm, with ∼5.90 nm bandwidth in the erbium laser, respectively. Figure 3(c) and (d) show the autocorrelation traces of the synchronized pulses, giving ∼8.75 ps pulse width in the ytterbium laser and ∼495 fs pulse width in the erbium laser. The output powers measured at 1 µm and 1.55 µm laser are ∼210.1 µW and 203.7 µW, respectively.

**Figure 4:**
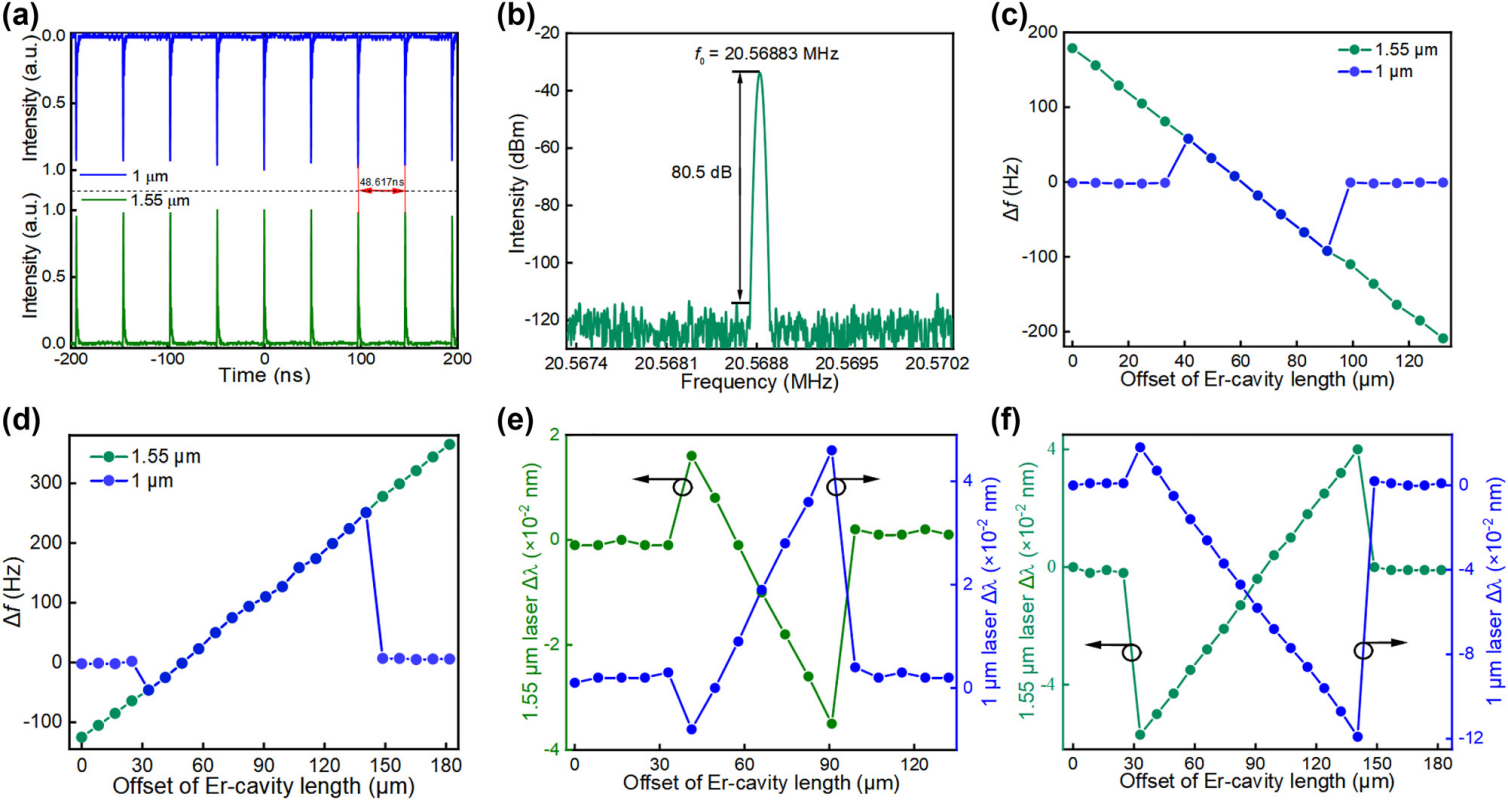
Synchronized laser repetition rates and peak wavelengths tunability as a function of the erbium cavity offset. (a) Synchronized laser pulse trains measured from ytterbium and erbium resonators; (b) radio frequency spectrum of the synchronized lasers; frequency offset of synchronized pulses as a function of cavity length difference when the erbium cavity length is increased (c) and decreased (d); peak wavelength shift versus cavity length difference when the erbium cavity length is increased (e) and decreased (f).

Synchronization of the two lasers can be characterized either in the time domain or frequency domain. Figure 4(a) and (b) are the mode-locked pulse trains and radio frequency spectrum measured when the synchronization is attained by tuning the delay in the erbium cavity. The synchronized frequency is ∼20.56883 MHz, corresponding to ∼48.617 ns pulse period. From the independent mode-locking, the fundamental repetition rate of the ytterbium laser is measured as ∼20.56880 MHz. After synchronisation, the ytterbium laser frequency slightly shifts to ∼20.56883 MHz, attributed to the refractive index change mainly caused by cross-phase modulation. Our further investigations focus on the frequency tuning characteristic when different erbium cavity offsets are applied. Here we define Δ*f* as the frequency offset to the fundamental repetition rate of the ytterbium laser, which is determined by a fixed cavity length. In the case that the erbium cavity length is shorter than the ytterbium cavity length, mode-locked synchronization forms when the two cavity lengths are getting closer by increasing the delay, as shown in Figure 4(c), where the ytterbium laser frequency offset is up to ∼60 Hz at an erbium cavity offset of ∼41.3 µm. Further decreasing the erbium laser repetition rate (i.e., increasing the erbium cavity length) from the synchronization initiation point, the synchronization repetition rate keeps going down to ∼20.56871 MHz, showing a tuning range of ∼150 Hz. On the contrary, the laser repetition rates are measured again when the erbium cavity length is decreased. Synchronization and non-synchronization are observed, but the synchronization frequency range is significantly enlarged to ∼300 Hz, as seen in Figure 4(d). This can be attributed to faster propagation of the erbium laser pulse in negative dispersion fiber, which causes more stable synchronization while the erbium cavity is shorter than the ytterbium cavity. Meantime, the tunability of the erbium laser cavity offset also leads to the peak wavelength shift in both lasers, which is due to the cross-phase modulation in anomalous dispersion [27, 28]. Here we define Δ*λ* as the peak wavelength shift to the original peak wavelength of the lasers before synchronization. Figure 4(e) plots the ytterbium and erbium laser peak wavelength shift as a function of the erbium cavity offset in the same tuning range as Figure 4(c). It can be clearly seen that the peak wavelengths present a hop when synchronization starts at ∼41.3 µm offset, and then shift to blue and red in the erbium and ytterbium lasers as the increase of offset. Noteworthy that the peak wavelengths return to their original values when the synchronization is detached at ∼99.12 μm offset, confirming that the shift is a result of cross-phase modulation. Peak wavelength shifts are measured as ∼0.051 nm in the erbium laser and ∼0.054 nm in the ytterbium laser, as shown in Figure 4(e). Interestingly, our followed reversal delay tuning experiment presents a similar wavelength tunability behavior, but with the erbium laser red-shifted and ytterbium laser blue-shifted instead. Experimental results are plotted in Figure 4(f), which shows ∼0.088 nm and 0.137 nm tuning of the erbium laser and the ytterbium laser, respectively. According to the relation of frequency and wavelength, the wavelength is blue-shifted (red-shifted) when the frequency is increased (decreased). Higher frequency wave of the pulse propagates faster in our negative dispersion cavity. Before tuning the delay, the erbium cavity is longer than the ytterbium cavity, the 1.55 µm pulse falls behind the 1 µm pulse when they propagate along the common arm. As the erbium cavity length approaches the ytterbium cavity by reducing the delay, the dual-wavelength pulses start to overlap in the time domain and pull each other, which causes an increase of the pulse repetition rate and blue-shift of the peak wavelength in the erbium laser, but reversal changes in the ytterbium laser. This pulling effect will result in the maximum overlap of the two pulses if the two cavity lengths are approximately equal.

## 5 Synchronization of the 1.55 µm laser with a 1.9 µm laser

In principle, the flat optical absorption spectrum of our DWCNT saturable absorber supports its availability for mode-locking of a wide range of wavelengths up to mid-infrared. As an important wave band for eye-safe applications and a potential alternative band in optical communication, thulium fiber lasers working around 2 µm have attracted increasing attention. The synchronization of dual-wavelength pulses enabled by our DWCNT sample can be extended to this wavelength region if proper fiber components are used. As a demonstration, we constructed a dual-wavelength laser with an erbium fiber cavity and a thulium fiber cavity in the same layout as the 1 µm and 1.55 µm laser setup (Figure 2). The same DWCNT saturable absorber synchronizing the 1 µm and 1.55 µm pulses is used again to mode-lock longer wavelengths. A 0.6 m erbium doped fiber (Liekki Er80-8/125) and a 2 m thulium doped fiber (OFS TMDF200) are utilized as gain media. The thulium doped fiber is pumped by a 1550 nm laser pre-amplified by an erbium-doped fiber amplifier through a 1550/1950 nm WDM. Another variable optical delay line is connected in the 1.55 µm cavity to adjust its optical length. The total length of each fiber cavity is approximately 11 m. An additional optical spectrum analyzer (APE waveScan) for the 1.9 µm laser is combined with the measurement equipment in the above experiment to analyze the laser performance.

**Figure 5:**
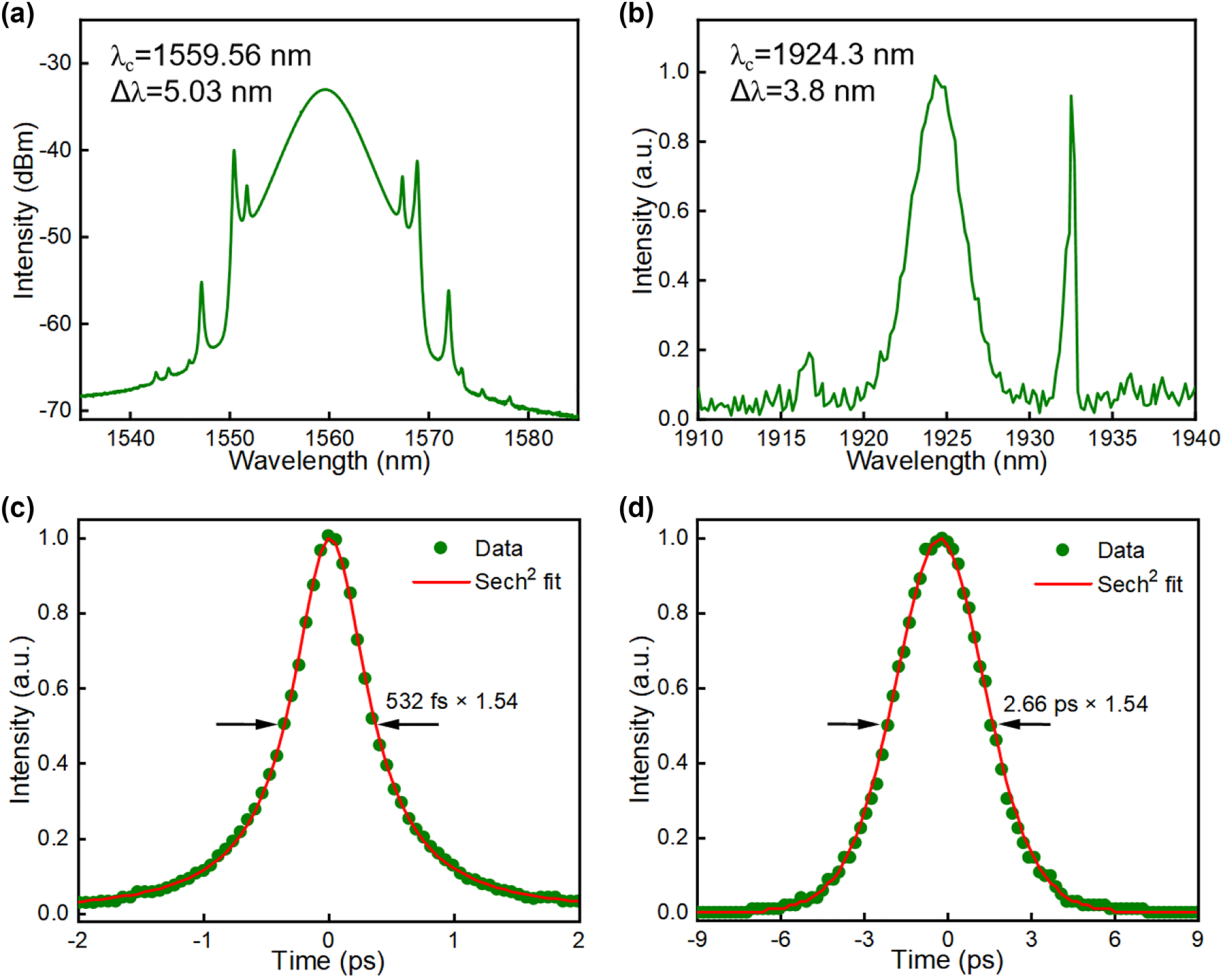
Synchronized lasers at 1.55 and 2 µm. (a) 1.55 µm erbium laser output soliton spectrum; (b) 2 µm thulium laser output soliton spectrum; (c) 1.55 µm erbium laser pulse autocorrelation trace; (d) 2 µm thulium laser pulse autocorrelation trace.

Stable mode-locking of the erbium laser and thulium laser is obtained independently when the pump powers reach 65.1 mW and 115.3 mW, respectively. Again, dual-wavelength pulse synchronization can be obtained when the erbium cavity length is tuned to approximately equal to the length of the thulium cavity. The mode-locked laser spectra remain the same in their independent operation, which centers at 1559.56 nm with 5 nm 3 dB bandwidth and 1924.3 nm with 3.8 nm 3 dB bandwidth in the erbium laser and thulium laser, respectively, as shown in Figure 5(a) and (b). Pulse autocorrelation traces measured in Figure 5(c) and (d) unveil pulse durations of 532 fs and 2.66 ps in the erbium laser and thulium laser.

**Figure 6:**
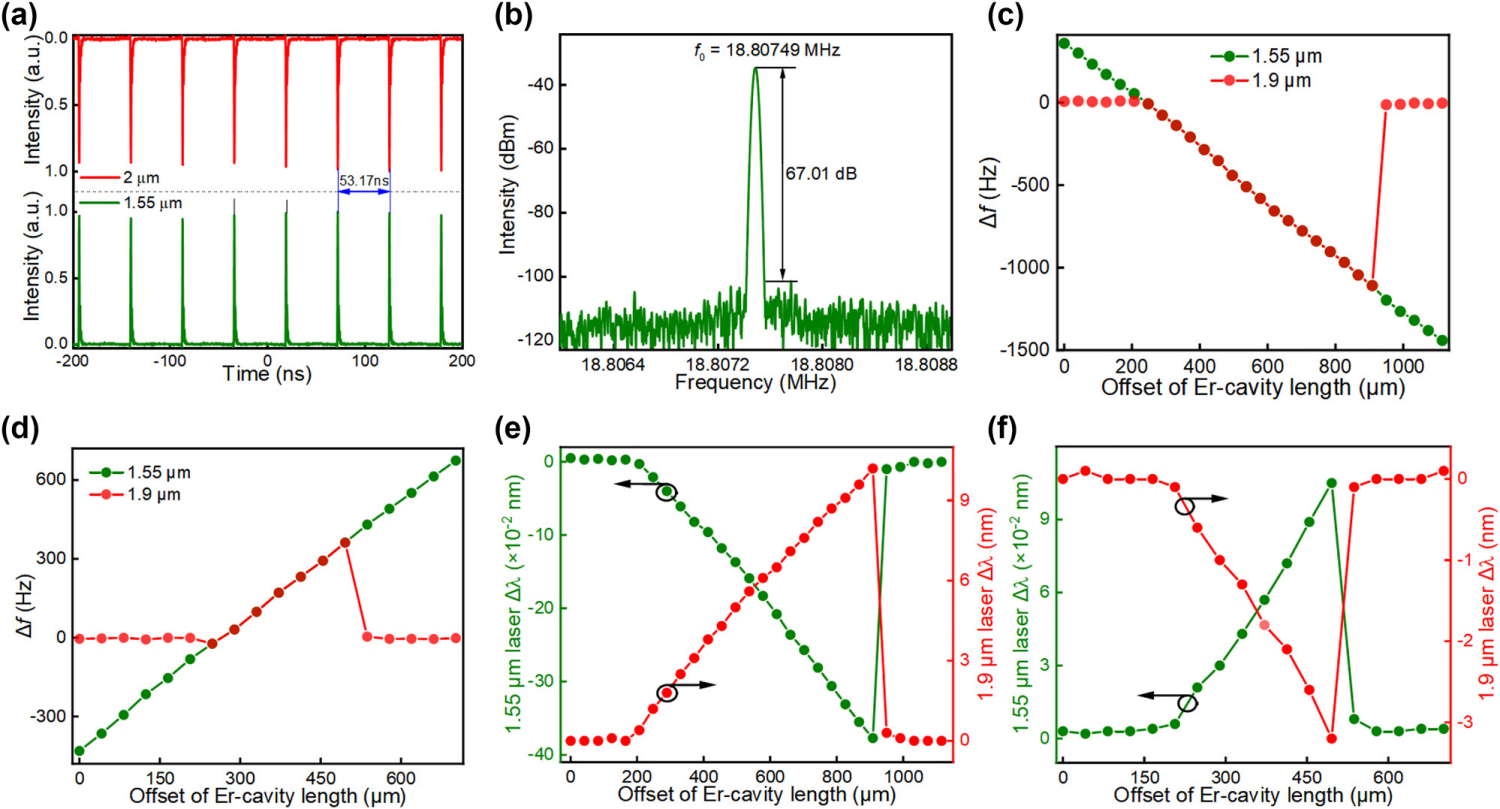
Synchronized laser repetition rates and peak wavelength tunability as a function of the Erbium cavity offset. (a) Synchronized laser pulse trains measured from the erbium and thulium resonators; (b) radio frequency spectrum of the synchronized lasers; frequency offset of synchronized pulses as a function of cavity length difference when the erbium cavity length is increased (c) and decreased (d); peak wavelength shift versus cavity difference when the erbium cavity length is increased (e) and decreased (f).

**Figure 7:**
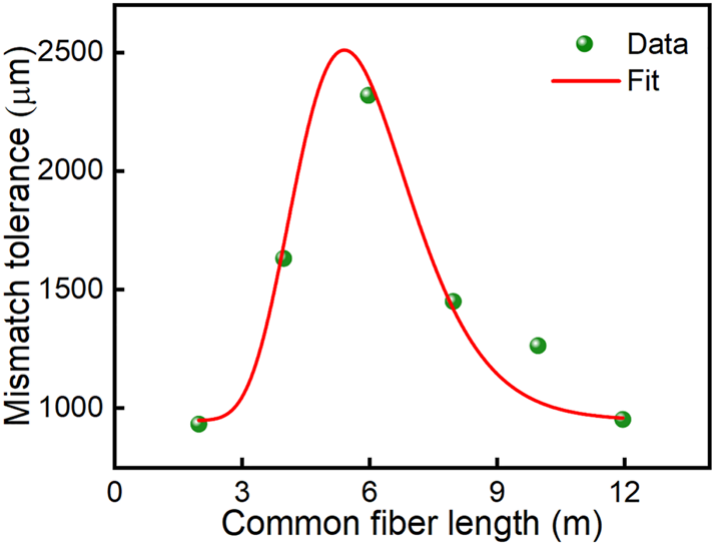
Mismatch tolerance as a function of the common fiber length.

Figure 6(a) and (b) are the synchronized pulse trains and radio frequency spectrum of the Erbium laser and thulium laser when their pump powers are ∼69.6 mW and 121 mW, respectively. The adjacent pulse spacing of the synchronized signal is ∼53.17 ns, corresponding to a repetition rate of ∼18.80749 MHz. The signal-to-noise contrast of >67 dB in Figure 6(b) verifies high stability in the synchronization mechanism. Again, we measure the dual-wavelength laser frequencies (repetition rates) as a function of the cavity offset by tuning the erbium cavity length in two opposite directions. The cavity mismatch tolerances reach ∼661 µm and 248 µm when the erbium cavity length increases and decreases, respectively, as shown in Figure 6(c) and (d). The tuning of synchronization frequency also accompanies the shifts of the peak wavelengths, which shows the same characteristic as the observation from the ytterbium and erbium lasers. However, the synchronization range of the erbium laser and thulium laser is much larger, which corresponds to the maximum frequency range of ∼1.1 kHz and causes the peak wavelength shift up to ∼0.38 nm and 10.2 nm for the 1559.56 nm and 1924.3 nm pulses, respectively (Figure 6(e)). The larger wavelength tuning may be attributed to the stronger nonlinear refractive index modulation as the pulse energy is much larger than that of the erbium and ytterbium lasers. In addition, we also study the influence of common fiber arm length on the synchronization performances, the experimental result is plotted in Figure 7. Via adjustment of the common fiber length from ∼2 m to 12 m, it can be seen that the maximum cavity mismatch tolerance is expanded up to ∼2360 μm at the optimal common fiber length of ∼6 m. This is a balanced consequence between the cross-phase modulation and group velocity dispersion.

To date, ultrafast pulse synchronization has been investigated in solid-state lasers, fiber lasers and their combined platforms. In solid-state lasers, Ti:sapphire has dominated the gain sources because of its broadband homogeneous gain spectrum and simplicity for self-mode-locking. Other gain media (such as Cr:forsterite and Nd:YVO_4_) are also options to extend the laser wavelength to a longer wavelength region. Particularly, fiber gain media with rare-earth doping further extend the synchronization laser from 1 µm to 2 µm, which is also the motivation for using DWCNT saturable absorber in this work. In terms of the synchronization technique, passive, active, and their hybrid method have been adopted as per the mode-locking mechanism. To give an overview of the performances of the previously studied dual-wavelength synchronization lasers, we summarize and list the key parameters of these lasers in Table 1 below. In comparison, our work demonstrates the synchronization of multiple wavelengths in ∼900 nm coverage with a single DWCNT saturable absorber, in which the largest cavity mismatch tolerance up to ∼2360 µm is achieved in the synchronization between 1559.56 nm and 1924.3 nm pulses.

**Table 1:** Synchronization of mode-locked lasers with different strategies.

Gain medium	Wavelength	Pulse width	Synchronization method	Repetition rate	Maximum mismatch	Refs
Ti:sapphire rod	790 nm, 760 nm	80 fs, 100 fs	Cross mode-locking	80.4 MHz	3 µm	[29]
Ti:sapphire rod	830 nm, 760 nm	30 fs, 64 fs	Self-mode-locking	76 MHz	1 µm	[30]
Ti:sapphire rod	755 nm, 750 nm	25 fs, 80 fs	Master-slave mode-locking	75.918 MHz	1.2 µm	[31]
Ti:sapphire rod	800 nm, 855 nm	50 fs, 109 fs	SESAM mode-locking with active feedback	119 MHz	/	[32]
Ti:sapphire rod	774 nm, 840 nm	55 fs, 65 fs	Dye mode-locking	85 MHz	/	[5]
Ti:sapphire rod	760 nm, 780 nm	105 fs, 80 fs	Cross mode-locking	79.5 MHz	3 µm	[6]
Ti:sapphire rod	853 nm, 767 nm	95 fs, 110 fs	Self-mode-lock	82 MHz	/	[7]
Ti:sapphire crystal, Cr:forsterite crystal	820 nm, 1250 nm	18 fs, 40 fs	SESAM and Kerr-lens mode-locking	78.8487 MHz	0.6 µm	[8]
Ti:sapphire crystal, Nd:YVO_4_ crystal	850 nm, 1064 nm	180 fs, 8 ps	Master-slave mode-locking	82.6 MHz	20 µm	[9]
Ti:sapphire crystal, Cr:forsterite crystal	833 nm, 1225 nm	30 fs	InGaAs mode-locking with active feedback	82 MHz	/	[10]
Ti:sapphire crystal, Cr:forsterite crystal	820 nm, 1230 nm	17 fs, 46 fs	Active (feedback)-passive hybrid mode-locking	101 MHz	/	[33]
Cr:forsterite crystal, Erbium fiber	1550 nm, 1250 nm	/	Master-slave mode-locking	40.75 MHz, 81. 5 MHz	9 µm, 12 µm	[34]
Ytterbium fiber, Erbium fiber	1060 nm, 1550 nm	2 ps, 1 ps	Master-slave mode-locking	40 MHz	140 µm	[17]
Ytterbium fiber, Erbium fiber	1040 nm, 1550 nm	13 ps, 200 fs	Master-slave mode-locking	29 MHz	20 µm	[18]
Erbium fiber	1540 nm, 1578 nm; 1557 nm	82 fs, 500 fs	Master-slave mode-locking	47.66 MHz	54 μm	[35]
Ytterbium fiber, Erbium fiber	1067.1 nm, 1535.48 nm	6.1 ps, 2.1 ps	Single-walled carbon nanotube mode-locking	6.54 MHz, 13.08 MHz	1400 µm	[36]
Ytterbium fiber, Erbium fiber	∼1030 nm, ∼1562 nm	∼110 fs, ∼70 fs	Master-slave mode-locking with active feedback	43.28 MHz	160 µm	[37]
Erbium fiber, Thulium fiber	1558.5 nm, 1938 nm	915 fs, 1.57 ps	Graphene mode-locking	20.5025 MHz	780 µm	[38]
Erbium fiber, Thulium fiber	1563.5 nm, 1931.9 nm	700 fs, 1.77 ps	Graphene mode-locking	12.905 MHz	/	[39]
Ytterbium fiber	1043 nm, 1057 nm	7.9 ps, 5.9 ps	Nonlinear polarization rotation mode-locking	38.1100 MHz	160 µm	[40]
Ytterbium fiber, Erbium fiber	1032.58 nm, 1558.48 nm	8.75 ps, 495 fs	DWCNT mode-locking	20.5688 MHz	∼500 µm	This work
Erbium fiber, Thulium fiber	1559.56 nm, 1924.3 nm	532 fs, 2.66 ps	DWCNT mode-locking	18.8075 MHz	∼2360 µm	This work

## 6 Design of synchronization of all three lasers at 1, 1.55, and 1.9 µm

**Figure 8:**
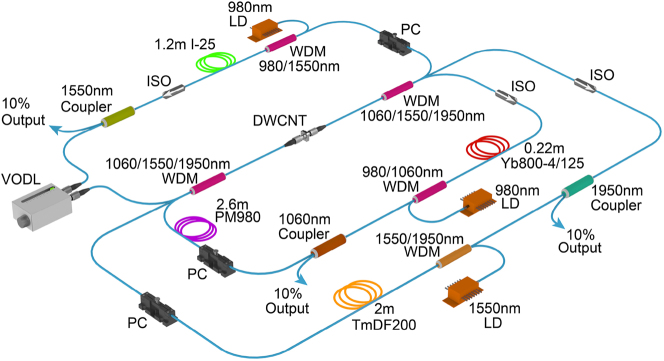
Conceptual diagram of the triple-wavelength synchronized fiber laser system.

Our work presented above demonstrates the synchronization of triple wavelengths in ∼900 nm coverage with two individual laser systems. To obtain triple-wavelength ultrafast laser synchronization from a single laser system would be more significant since it may have more potential applications, like high-energy femtosecond pulse emission and multi-photon pump-probe investigations. Here we propose a conceptual diagram of the triple-wavelength synchronized fiber laser system in Figure 8, which is an upgrade based on Figure 2. The ytterbium and erbium cavities keep the same configurations depicted in Figure 2, and the thulium cavity uses the same configuration described in Section 5. The total length of the thulium cavity is modified the same as the ytterbium cavity. Two triple-wavelength WDMs replace the original dual-wavelength WDMs at the two ends of the common arm to couple three ring resonators. Cross-phase modulation induced pulse attraction leads to the synchronization of all three lasers at 1, 1.55, and 1.9 µm. Further, multi-wavelength (more than triple wavelengths) synchronization could also be achieved with a broader coverage if multi-wavelength WDMs are applied at the two ends of the common arm. Since passive synchronization can only be achieved with optical path differences of the cavities on the order of microns, more optical variable delay lines work at different wavelengths and could be applied in the cavities to tune the optical lengths precisely and promote multi-wavelength synchronization.

Although the triple-wavelength synchronized fiber laser system proposed here is still a conceptual methodology, which is limited by existing fabrication technology of multi-wavelength WDM, the development of optical components will certainly enable the system to be implemented in the near future for applications.

## 7 Conclusions

In conclusion, we demonstrate the synchronization of a 1.55 µm laser with a 1 µm laser and a 1.9 µm laser, respectively, by using the same DWCNT film as a saturable absorber. The two synchronization lasers adopt the same cavity layout with two ring fiber resonators coupled in a shared section, where the broadband DWCNT saturable absorber is placed to start the mode-locking of different wavelengths. Our results indicate that the tuning range of repetition rate and wavelength are strongly relevant to the common arm length, which can be attributed to the interaction between cross-phase modulation and cavity dispersion. Via the optimization of common arm fiber length, we obtain the largest synchronization cavity mismatch tolerance up to ∼2360 µm in the erbium and thulium fiber laser. Also, we present a creative concept to realize multi-wavelength synchronization from a single laser system. Our work finds a simple and compact solution for the construction of dual-wavelength synchronized fiber lasers operating at typical technical wavelengths from near-infrared to the mid-infrared range, which will facilitate a number of application demands where pulse synchronization is indispensable.
